# Clinicopathologic Features of Metaplastic Breast Carcinoma: Experience From a Tertiary Cancer Center of North India

**DOI:** 10.7759/cureus.28978

**Published:** 2022-09-09

**Authors:** Vineeth V Damera, Zachariah Chowdhury, Mayank Tripathi, Rupesh Singh, Ravinder K Verma, Meenal Jain

**Affiliations:** 1 Surgical Oncology, Mahamana Pandit Madan Mohan Malaviya Cancer Centre & Homi Bhabha Cancer Hospital (Tata Memorial Hospital), Varanasi, IND; 2 Pathology, Mahamana Pandit Madan Mohan Malaviya Cancer Centre & Homi Bhabha Cancer Hospital (Tata Memorial Hospital), Varanasi, IND

**Keywords:** postoperative radiation therapy, breast conservation surgery, modified radical mastectomy (mrm), squamous cell carcinoma (scc), hormone receptors in breast cancer, triple-negative breast carcinoma, breast histopathology, spindle cell metaplastic breast cancer, breast invasive carcinoma

## Abstract

Introduction

Metaplastic breast cancer (MBC) is a rare malignancy that accounts for < 1% of all breast cancers. The aim of this study is to evaluate the clinicopathologic characteristics of MBC patients treated at a tertiary cancer center.

Materials and methods

In this study, the authors retrospectively analyzed the prospectively maintained data of MBC patients treated at a tertiary cancer care center in North India between January 2019 and July 2022.

Results

A total of 28 MBCs were identified. The median age of presentation was 47 years (range 27-81 years). Seventeen patients (60.7%) presented with clinical T3/T4 disease, and axillary nodal involvement was detected in 11 patients (39.3%) at presentation. Two patients had metastatic disease at presentation. A preoperative diagnosis of MBC on core biopsy was attained in five patients (17.9%), and the most common histologic subtype was sarcomatoid carcinoma. Triple-negative receptor status was observed in 15 patients (53.6%). Six patients (21.4%) underwent upfront breast conservation surgery and another six (21.4%) upfront mastectomy. Thirteen patients (46.4%) underwent mastectomy following neoadjuvant therapy. Definitive axillary nodal metastasis was found in eight patients (32%). Following neoadjuvant chemotherapy, five patients (35.7%) had stable disease, disease progression was evident in five patients (35.7%), partial response in four patients (28.6%), and no patient evinced complete response. Adjuvant postoperative radiation therapy was administered in 16 patients (57.1%). At a median follow-up of 13.2 months (range 4-26 months), 16 patients (57.1%) were alive with no evidence of disease, one patient (3.6%) was alive with disease, nine patients (32.1%) died of disease, and two patients (7.2%) died of other causes. One patient suffered from locoregional recurrence and nine patients developed distant metastasis.

Conclusion

MBC is an infrequent entity among breast carcinomas in India, which is similar to the reports of MBC worldwide. The diagnosis of MBC is difficult and requires the use of immunohistochemistry. Most of the cases in our study presented with a larger tumor size; however, they displayed a relatively lower incidence of nodal involvement as well as hormone receptor negativity. Being a rare and heterogeneous disease, large-scale studies are essential for better understanding and management of these tumors.

## Introduction

Constituting < 1% of all invasive breast cancers, metaplastic breast cancer (MBC) is a seldom encountered malignancy [1,2]. The term “metaplastic carcinoma” was first described by Huvos and colleagues in 1973 [3]. It encompasses a heterogeneous group of tumors characterized by the metaplastic transformation of the glandular epithelium into squamous epithelium or mesenchymal elements such as spindle, chondroid, osseous, and rhabdoid differentiation [4]. The clinical presentation of patients with MBC is larger tumor size, higher grade and stage, more hormone receptor-negative tumors with less frequent involvement of regional nodes, and a higher likelihood of distant metastasis in comparison to classical breast invasive carcinoma [5]. Patients with MBC tend to have a worse outcome when compared with triple-negative breast cancer (TNBC) [6]. Because of the rarity of this disease and unfamiliar biologic characteristics of MBC, this study was undertaken to evaluate MBC with regard to its clinicopathologic characteristics, its response to multidisciplinary therapeutic modalities, and its outcome at a tertiary cancer center in North India.

## Materials and methods

A retrospective analysis of the prospectively maintained data of MBC patients managed at a tertiary cancer care center in North India from January 2019 to July 2022 was undertaken, following the guidelines of the institutional ethics committee. The authors evaluated the data with respect to patient age, gender, tumor size, lymph node status, clinical stage, histologic grade, receptor status (estrogen receptor (ER), progesterone receptor (PR), and Her2/neu), ductal carcinoma in situ (DCIS) component, type of surgical procedure, chemotherapy (adjuvant, neoadjuvant, palliative chemotherapy) and/or radiotherapy (adjuvant/palliative) and outcome. The treatment protocol of MBC at our institute follows the same principles of invasive carcinoma of the breast. Patients with early disease (T1/T2 and N0/N1, T3N0) were offered upfront surgery followed by adjuvant treatment (chemotherapy, radiotherapy, and hormonal treatment). Patients with locally advanced disease (T3/T4 or N2/N3) or those who needed tumor downstaging for breast conservative surgery were treated with neoadjuvant chemotherapy (NACT) followed by the appropriate surgery and then adjuvant radiotherapy (RT) ± hormonal treatment (according to the hormonal receptor status). At our center, taxane-based NACT is preferred in patients with MBC. The response following NACT was evaluated by using the response evaluation criteria in solid tumors (RECIST).

## Results

A total of 28 MBC cases were identified based on histopathology reports, and tumor subtyping was done according to the latest 2019 WHO classification. The incidence was 1.14% of all invasive breast carcinomas presenting at our center during the study period. The clinicopathological characteristics of the 28 cases have been detailed in Table 1 and Table 2.

**Table 1:** Clinicopathologic features of 28 patients with metaplastic breast carcinoma NACT: neoadjuvant chemotherapy. CT: chemotherapy. RT: radiation therapy. E: estrogen receptor. P:progesterone receptor. H: Her2/neu. Y: Yes. N: No. SCC: squamous cell carcinoma. HGAS: high-grade adenosquamous carcinoma. SpC: spindle cell carcinoma. MCHMD: metaplastic carcinoma with heterologous mesenchymal differentiation. SC: sarcomatoid carcinoma. PR: partial response. SD: stable disease. PD: progressive disease. SMAC: simple mastectomy with axillary clearance. AS: axillary sampling. LM: liver metastasectomy. SCFC: supraclavicular fossa clearance. RM: radical mastectomy. LDF: latissimus dorsi flap. TAF: thoracoabdominal flap. AC: axillary clearance. PCT: palliative chemotherapy. PRT: palliative radiation therapy. DM: distant metastasis. LRR: locoregional recurrence. DOD: died of disease. NED: no evidence of disease. DOOC: died of other causes. AWD: alive with disease.

SL NO	AGE (yrs)	SEX	cTNM	Histologic subtype	Hormone profile	NACT & response	Surgery	pTNM	CT	RT	Recurrence	Followup (months)	Final status
1.	27	F	T4N0M1	SCC	E-P+H+	N	N	---	PCT	N	---	06	DOD
2.	39	F	T2N1M0	HGAS	E+P-H-	Y, Stable	SMAC	ypT2N3a	Y	Y	N	26	NED
3.	37	F	T4N0M0	HGAS	E-P-H+	Y, PR	SMAC	ypT4bN0	Y	N	Y, DM	13	DOD
4.	28	F	T2N0M0	SpC	TNBC	N	BCS+AS	pT2N0	Y	Y	N	25	NED
5.	47	F	T2N0M0	MCHMD	TNBC	Y, SD	SMAC	pT2N0	Y	Y	Y, DM	25	DOD
6.	38	F	T2N0M0	SC	TNBC	N	MRM+AS	pT2N0	Y	N	N	25	NED
7.	30	F	T4N1M0	SCC	E+P-H-	Y, PD	SMAC	ypT4aN1a	Y	Y	N	06	NED
8.	38	F	T3N1	SCC	E+P-H-	Y, PD	SMAC	ypT4bN1a	Y	N	Y, DM	04	DOD
9.	57	F	T4N0	SC	TNBC	Y, SD	SMAC	ypT3N0	Y	Y	N	12	NED
10.	62	F	T3N1	SC	E+P+H-	Y, PR	SMAC+LM	ypT2N2aM1	Y	Y	N	10	NED
11.	62	F	T4N1	HGAS	E+P-H-	Y, PR	SMAC	ypT3 N0	Y	Y	N	09	NED
12.	54	F	T2N0	SC	TNBC	N	BCS+AS	pT2N0	Y	Y	N	11	NED
13.	32	F	T2N0	SC	TNBC	Y, SD	SMAC	ypT2N0	Y	N	N	16	NED
14.	50	M	T3N1M1	MCHMD	E+P+H-	N	N	---	PCT	PRT	---	17	DOD
15.	67	F	T4bN3c	MCHMD	E+P+H-	Y, PR	SMAC+Left SCFC	ypT3N1	Y	N	---	---	DOOC
16.	42	F	T4N0	MCHMD	TNBC	Y, PD	SMAC	ypT4N0	Y	Y	Y, DM	11	DOD
17.	44	F	T4N0	SC	TNBC	Y, SD	SMAC	ypT4N0	Y	Y	Y, DM	12	DOD
18.	47	F	T4N2M0	HGAS	E-P-H+	Y, PD	N	---	Y	N	---	12	DOD
19.	43	F	T3N0	MCHMD	TNBC	N	MRM+AS	pT3N0	Y	N	N	25	NED
20.	47	F	T4N0	HGAS	TNBC	N	RM+LDF	pT4N0	Y	PRT	Y, DM	08	DOD
21.	73	F	T3N0	SC	E+P+H-	N	MRM+AS	pT3N0	Y	Y	N	05	NED
22.	43	F	T4bN1	SC	E-P+H-	Y, PD	SMAC+TAF	pT4bN1a	Y	N	N	---	DOOC
23.	36	F	T2N0	SC	TNBC	N	BCS+AS+LD	pT2N1a	Y	Y	N	07	NED
24.	30	F	T4N1	SC	TNBC	N	RM	pT3N1a	Y	Y	N	07	NED
25.	58	F	T2N0	SC	TNBC	N	BCS+AS	pT2N0	Y	Y	N	10	NED
26.	47	F	T2N1	SC	TNBC	N	BCS+AC	pT2N0	Y	Y	N	10	NED
27.	81	F	T2N0	SCC	E+P-H-	N	MRM+AC	pT2N0	N	N	N	08	NED
28.	32	F	T2N0	SC	TNBC	N	BCS+AS	PT2N0	Y	Y	Y, LRR	24	AWD

**Table 2:** Characteristics of MBC patients according to several evaluated parameters (n=28) MBC: metaplastic breast carcinoma. T: tumor. N: nodal. M: metastasis. IBC NST: invasive breast carcinoma, no special type. HMD: heterologous mesenchymal differentiation. DCIS: ductal carcinoma in situ. BCS: breast conservation surgery. NACT: neoadjuvant chemotherapy

Sl No	Parameters	Number (n)	Percentage (%)
1.	Age		
	> 40 years	11	39.3
	< 40 years	17	60.7
2.	Sex		
	Female	27	96.4
	Male	01	3.8
3.	Clinical T Stage		
	T1/T2	11	39.3
	T3/T4	17	60.7
4.	Clinical N Stage		
	N0	17	60.7
	N1	11	39.3
5.	M Stage		
	M0	26	92.9
	M1	02	7.1
6.	Stage at presentation		
	Early (T2 N0/N1, T3N0)	12	42.9
	Locally advanced (T3/T4, N+)	14	50
	Metastatic	02	7.1
7.	Diagnosis on core biopsy		
	Diagnosed on core biopsy	05	17.9
	Suspicious of MBC on core biopsy	04	14.3
	Misdiagnosed as IBC NST	14	50
	Misdiagnosed as Phyllodes tumor	04	14.3
	Could not be categorized (Poorly differentiated malignancy)	01	3.6
8.	Histopathologic subtype		
	Sarcomatoid carcinoma (Biphasic)	13	46.4
	High grade adenosquamous carcinoma	05	17.9
	Pure squamous cell carcinoma	04	14.3
	MBC with heterologous mesenchymal differentiation	05	17.9
	Spindle cell carcinoma	01	3.6
9.	Lymphovascular invasion		
	Yes	05	17.9
	No	23	82.1
10.	In situ component (DCIS)		
	Present	03	10.7
	Absent	25	89.3
11.	Hormone Receptor status		
	Positive	11	39.3
	Negative	17	60.7
12.	Her2/neu status		
	Positive	03	10.7
	Negative	25	89.3
13.	Triple negative MBC		
	Yes	15	53.6
	No	13	46.4
14.	Type of Surgery		
	Upfront Breast Conservation Surgery	06	21.4
	Upfront Mastectomy	06	21.4
	Mastectomy following NACT	13	46.4
	No surgery in view of metastasis	03	10.7
15.	Pathologic Tumor stage		
	pT0	0	0
	pT1	0	0
	pT2	12	48
	pT3	06	24
	pT4	07	28
16.	Pathologic Nodal stage		
	pN0	17	68
	pN1	06	24
	pN2	01	04
	pN3	01	04
17.	Chemotherapy		
	Yes	27	96.4
	No	01	3.6
18.	Radiation therapy (RT)		
	Adjuvant RT	16	57.1
	Not given	10	35.8
	Palliative	02	7.1
19.	Hormonal therapy		
	Yes	09	32.1
	No	19	67.9
20.	Clinical response to NACT		
	Complete response	0	0
	Partial response	04	28.6
	Stable disease	05	35.7
	Progressive disease	05	35.7
21.	Locoregional Recurrence		
	Yes	01	3.6
	No	27	96.4
22.	Distant metastasis		
	Metastasis at presentation	02	7.1
	Metastasis on NACT	02	7.1
	Metastasis after treatment	05	17.9
	No distant metastasis	18	64.3
23.	Site of distant metastasis		
	Lung and/or pleura	04	44.5
	Lung and liver	01	11.1
	Lung/pleura and bone	01	11.1
	Lung and brain	03	33.3
24.	Patient status		
	Alive with disease	01	3.6
	No evidence of disease	16	57.1
	Died of disease	09	32.1
	Died of other causes	02	7.2

**Figure 1:**
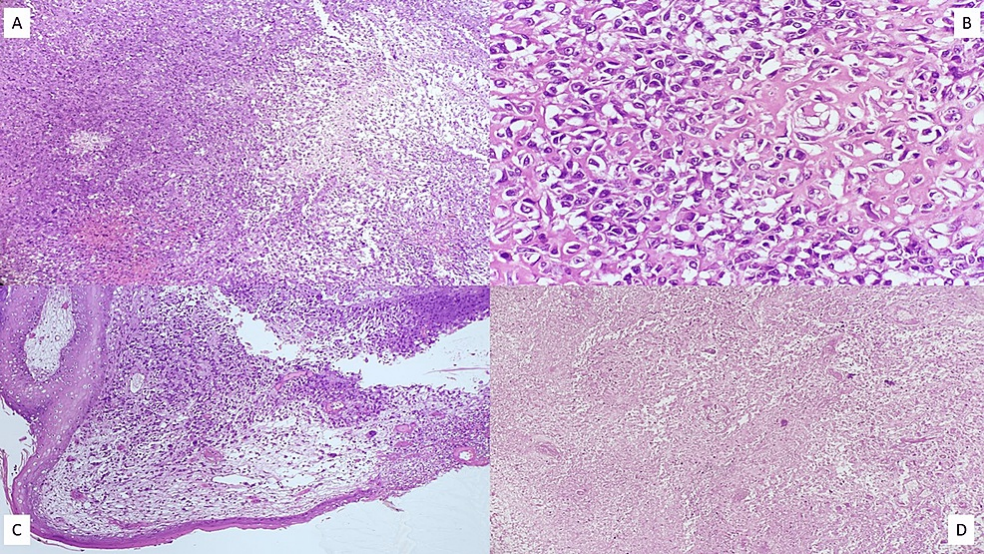
Photomicrographs of the histopathology of a case of MBC (sarcomatoid carcinoma) with epithelial and sarcomatoid areas (H&E, (A) 20X), osteoid matrix (H&E, (B) 40X), skin ulceration (H&E, (C) 20X), and necrosis (H&E, (D) 20X) MBC: metaplastic breast carcinoma

**Figure 2:**
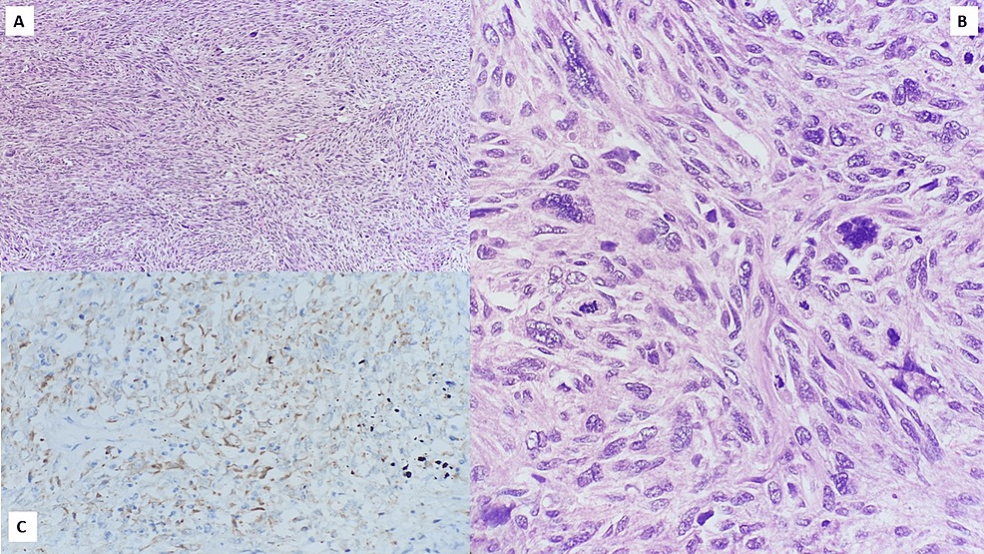
Photomicrographs of the histopathology of a case of MBC (sarcomatoid carcinoma) (H&E, (A, 10X), (B, 40X)) demonstrating faint to moderate positivity for PanCK ((C), 20X). MBC: metaplastic breast carcinoma. PanCK: pancytokeratin

**Figure 3:**
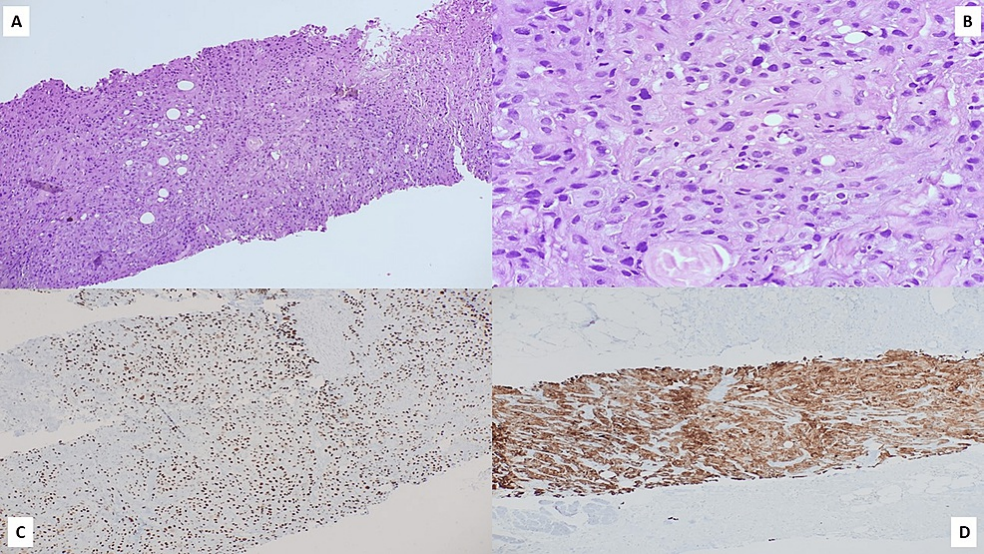
Photomicrographs of the histopathology of a case of MBC (squamous cell carcinoma) (H&E, (A, 10X), (B, 40X)) exhibiting positivity on immunohistochemistry for p40 ((C), 10X) and CK7 ((D), 10X) MBC: metaplastic breast carcinoma

The median age at diagnosis was 47 years (range 27-81 years) with only one male patient in our data set. Clinical examination revealed T3/T4 disease in 17 patients (60.7%) and involvement of axillary lymph nodes in 11 patients (39.3%). Fourteen patients (50%) displayed locally advanced breast cancer (cT3/T4N1, N2, N3, or N2/N3 with any T), and two patients (7.1%) harbored distant metastasis on presentation. On core needle biopsy, MBC was misdiagnosed as invasive breast carcinoma, no special type (IBC NST) in 14 patients (50%), and phyllodes tumor in four patients (14.3%). In these patients, a histopathologic diagnosis of MBC was made on the resection specimens after the surgery. Preoperative diagnosis of MBC on core needle biopsy was achieved in five patients (17.9%). In our series, the most common pathological subtype of MBC was biphasic sarcomatoid carcinoma (SC), which was conspicuous in 13 patients (46.4%) (Figures 1, 2). Five patients (17.9%) disclosed MBC with heterologous mesenchymal differentiation (MCHMD) while another five patients (17.9%) exhibited high-grade adenosquamous carcinoma (HGAS). Pure squamous cell carcinoma (SCC) was discernible in four patients (14.3%) (Figure 3), whereas spindle cell carcinoma (SpCC) was observed only in a single patient (3.6%).

Hormone receptor positivity (ER, PR positive) was identified in 11 patients (39.3%), Her2/neu positivity was encountered in three patients (10.7%), out of which two patients revealed exclusive Her2/neu positivity (7.1%). An unexpected finding was the lower percentage of triple negativity for hormone receptors, evident in 15 patients (53.6%). Comparing triple negative MBCs (TNMBC) with Non-TNMBCs (NTNMBC) {Table 3}, sarcomatoid carcinomas exhibited a higher percentage of TNMBC (66.7%) versus the other histologic subtypes, progressive disease was identified more in the NTNMBC subgroup (44.5% in NTNMBC vs 20% in TNMBC), while distant metastasis was almost similar in both the subgroups. The TNMBC subgroup revealed a higher percentage of disease-free status (66.7%) juxtaposed with the NTNMBC subgroup (46.2%).

**Table 3:** Comparison of triple-negative (TN) MBC with non-TNMBC (NTNMBC) MBC: metaplastic breast carcinoma. NTNMBC: non-triple-negative MBC. CR: complete response. PR: partial response. SD: stable disease. PD: progressive disease. MRM: modified radical mastectomy. RM: radical mastectomy. BCS: breast conservation surgery.

Sl No	Features	TNMBC (n=15)	NTNMBC (n=13)
1.	Histologic subtype		
	SC	10	3
	MCHMD	3	2
	SpC	1	0
	HGAS	1	4
	SCC	0	4
2.	NACT & Response	5 Patients had received NACT	9 Patients had received NACT
	CR	0	0
	PR	0	4
	SD	4	1
	PD	1	4
3.	Surgery		
	MRM & RM	4	2
	BCS	6	0
4.	Recurrence		
	Locoregional Recurrence	1	0
	Distant Metastasis		
	Metastasis at presentation	0	2
	Metastasis on NACT	0	1
	Metastasis after treatment	4	2
5.	Final status		
	Alive with disease (AWD)	1	0
	No evidence of disease (NED)	10	6
	Died of disease (DOD)	4	5
	Died of other causes (DOOC)	0	2

Thirteen patients (46.4%) underwent a mastectomy and axillary clearance following NACT. Upfront mastectomy (modified radical mastectomy in four cases and radical mastectomy in two cases) was performed in six (21.4%) patients while breast conservative surgery (BCS) was possible in six cases (21.4%). Three patients did not undergo surgery, as there was evidence of distant metastasis on positron-enhanced tomography/computed tomography (PET/CT) imaging. Twelve patients (48%) manifested pathological T2 (pT2) disease, six patients (24%) had pathological T3 (pT3) disease, and seven patients (28%) divulged pathological T4 (pT4) disease. None of the patients bore pT0 and pT1 disease. Lymphovascular invasion (LVI) was ascertained in five patients (17.9%), and the DCIS component was apparent in only three patients (10.3%) in our cohort.

**Figure 4:**
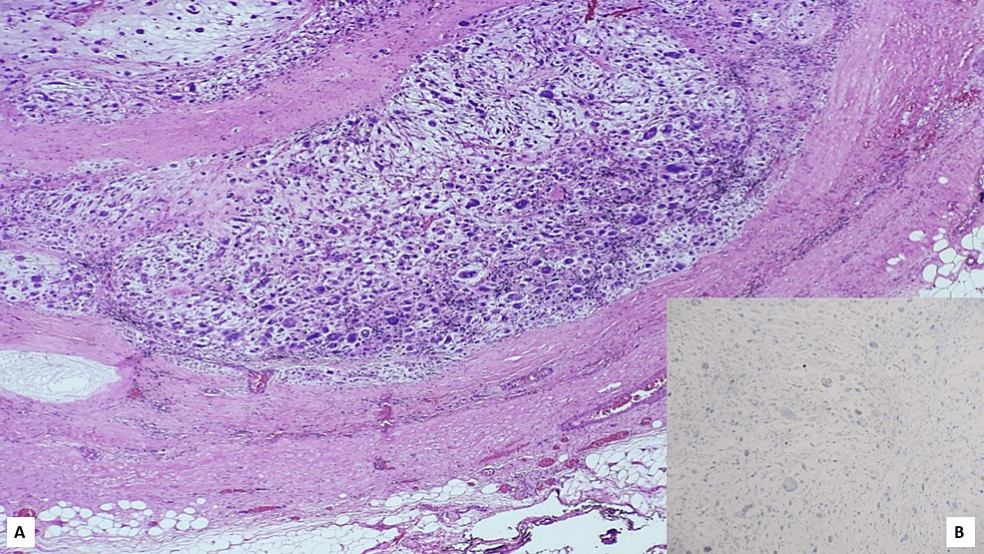
Photomicrographs of the lymph node metastasis of a case of MBC (sarcomatoid carcinoma) The histopathology demonstrates large pleomorphic tumor cells in a myxoid stroma (H&E, (A, 40X), showing negativity for PanCK (B, 40X)). MBC: metaplastic breast carcinoma. PanCK: pancytokeratin

Axillary sampling was executed in nine patients (36%) while axillary clearance was conducted in 16 patients (64%). Node negative (pN0) emerged in 17 of 25 patients (68%) on final histopathology. Among those with metastatic axillary nodes, six patients (24%) had pN1 disease, one each (4%) pN2 and pN3 disease. Corresponding to the histologic subtype, axillary lymph nodal metastasis was observed in 30.8% (4/13) of SC, 50% of SCC (2/4), and 20% each in HGAS (1/5) and MCHMD (1/5) (Figure 4).

Chemotherapy was administered for neoadjuvant, adjuvant, and palliative purposes in 14, 25, and two patients, respectively. Following NACT, five patients bore stable disease, five patients suffered disease progression, four patients had a partial response and none expressed complete response. Adjuvant postoperative RT was administered in 16 patients (57.1%). At the last follow-up, there was one locoregional recurrence and nine patients endured distant metastasis. At a median follow-up of 13.2 months (range 4-26 months), 16 patients (57.1%) were alive with no evidence of disease (NED), one patient (3.6%) was alive with disease (AWD), nine patients (32.1%) died of disease (DOD), and two patients (7.2%) died of other causes (DOOC). The oncological causes of death were brain metastasis in some patients and lung metastasis in others.

## Discussion

MBC is a heterogeneous group of invasive breast carcinomas characterized by the transformation of part or all of its glandular component into a non-glandular or metaplastic component, such as squamous cells and/or mesenchymal-looking elements, including but not restricted to the spindle, osseous, and chondroid cells [7]. The latest 2019 World Health Organization (WHO) classification of breast tumors [8] classifies MBC on the basis of histological patterns into:

a. Epithelial-only carcinomas, which include low-grade adenosquamous carcinoma (LGAS), high-grade adenosquamous carcinoma (HGAS), and squamous cell carcinoma (SCC).

b. Sarcomatoid carcinoma, which is divided into i. Pure (monophasic), which includes spindle cell carcinoma (SpCC) and metaplastic carcinoma with heterologous mesenchymal differentiation (MCHMD), and ii. Biphasic, which includes sarcomatoid carcinoma (SC) with both epithelial and sarcomatoid areas

c. Mixed metaplastic carcinoma (MMC), which includes i. A mixture of different metaplastic elements, and ii. Metaplastic and conventional adenocarcinomatous components [8].

Table 4 shows the WHO classification of breast tumors.

**Table 4:** 2019 World Health Organization (WHO) classification of breast tumors Source: [8]

Sl No	Broad heading	Subheading	Additional typing
1.	Epithelial tumors	a) Benign epithelial proliferations & precursors	
		b) Adenosis & benign sclerosing lesions	
		c) Adenomas	
		d) Epithelial myoepithelial tumors	
		e) Papillary neoplasms	
		f) Non-invasive lobular neoplasia	
		g) Ductal carcinoma in situ	
		h) Invasive breast carcinoma	i) Invasive breast carcinoma of no special type
			ii) Microinvasive carcinoma
			iii) Invasive lobular carcinoma
			iv) Tubular carcinoma
			v) Cribriform carcinoma
			vi) Mucinous carcinoma
			vii) Mucinous cystadenocarcinoma
			viii) Invasive micropapillary carcinoma
			ix) Carcinoma with apocrine differentiation
			x) Metaplastic carcinoma
		i) Rare & salivary gland-type tumors	
		j) Neuroendocrine neoplasms	
2.	Fibroepithelial tumors & hamartomas	a) Hamartoma	
		b) Fibroadenoma	
		c) Phyllodes tumor	
3.	Tumors of the nipple	a) Syringomatous tumor	
		b) Nipple adenoma	
		c) Paget disease of the breast	
4.	Mesenchymal tumors	a) Vascular tumors	
		b) Fibroblastic & myofibroblastic tumors	
		c) Peripheral nerve sheath tumors	
		d) Smooth muscle tumors	
		e) Adipocytic tumors	
		f) Other mesenchymal tumors & tumor-like conditions	
5.	Hematolymphoid tumors	Lymphoma	
6.	Tumors of the male breast	a) Gynaecomastia	
		b) Carcinoma in situ	
		c) Invasive carcinoma	
7.	Metastases to the breast		
8.	Genetic tumor syndromes		

The incidence of MBCs in our study (1.14%) was slightly higher than in the standard literature. The median age at diagnosis for patients with MBC reported in the literature ranges from 46-59 years [9,10]. In our series, the median age of presentation was 47 years. There was one male patient in our case series, the rest were females. Although there is a female preponderance, male MBC patients have rarely been described in the literature [11]. Clinically and radiologically, it presents similarly to other breast cancers [12]; albeit MBC patients usually manifest with larger tumor size, higher grade, and stage, higher incidence of hormone receptor-negativity, less frequent involvement of regional nodes, and a higher likelihood of distant metastasis in comparison to classical invasive breast carcinoma [5]. In our series, 14 patients (50%) experienced locally advanced disease T3/T4 disease and two patients (7.1%) were metastatic at presentation. Even though the lymphatic spread is less common, the reported incidence of nodal spread varies from around 27-64% [9,13] in different studies. Axillary nodal involvement was recognized in eight patients (32%) in our study. LVI was present in five patients (17.9%), which is lower than that chronicled by Rakha et al. (21%) and Erjan et al. (27.2%) [14,15]. The prevalence of DCIS in MBC is less compared to IBC NST, which is associated with 80% DCIS. Rakha et al. documented the DCIS component in 42% of their cohort while Erjan et al. registered this finding in 39.5% of cases [14,15]. The aforementioned observation is significantly higher when compared to our study (10.3%). Various parameters unraveled in our study have been collated with the standard literature in Table 5.

**Table 5:** Comparison of our study with previous studies NR: not reported. LRR: locoregional recurrence.DM: distant metastasis. PD: progressive disease. AWD: alive with disease. DOD: died of disease. NED: no evidence of disease.

Sl No	Study	No of cases	Median age (years)	cT stage -%	Clinical nodal positivity (%)	pT stage- %	Pathological nodal positivity (%)	Triple-negative receptor status (%)	Follow-up (months)	Outcome	Patient status
1.	Esbah et al. [9]	14	45.5	cT1/T2- 42.8	NR	pT1- 0	64.3	71.4	52	LRR- 7.1%	PD- 50%
				cT3/T4- 57.2		pT2- 30.8				DM- 57.1%	Death- 35.7%
						pT3- 61.5					
						pT4- 7.7					
2.	Hasbay et al. [16]	38	55.34	NR	NR	pT1- 7.9	49	78.9	34	LRR- NR	Alive- 81.5%
						pT2- 52.6				DM- 28.9%	Dead- 18.5%
						pT3- 10.5					
						pT4- 7.9					
3.	Samoon et al. [17]	42	54	cT1/T2- 73.8	45.2	NR	53.1	38.1	34	LRR- 5.2%	AWD- 7.7%
				cT3/T4- 26.2						DM- 24.32%	DOD- 17.9%
											NED- 69.2%
4.	Erjan et al. [15]	81	48	NR	NR	pT1/T2- 64.2	34.6	67.9	54	LRR- 18.5%	AWD- 4.9%
						pT3/T4- 35.8				DM- 34.6%	DOD- 30.9%
											NED- 55.6%
5.	Current study	28	47	cT1/T2- 39.3	39.3	pT0/T1- 0	32	53.7	13	LRR- 3.6%	AWD- 3.6%
				cT3/T4- 60.7		pT2- 48				DM- 32.1%	DOD- 32.1%
						pT3- 24					NED- 57.1%
						pT4- 28					

It is difficult to establish a histopathological diagnosis of MBC on core biopsy. A study conducted by Park et al. showed that preoperative diagnosis of MBC on core biopsy was possible only in 4.2% of cases [18]. In our series, we could make a confident diagnosis of MBC in 17.9% of patients based on core biopsy; the rest were detected only on the final histopathology of the resected specimen after definitive surgery. The diagnosis of MBC on core biopsy is problematic when there is spindle cell morphology without an epithelial or DCIS component. To confidently diagnose MBC on core biopsy requires a high degree of pathologic acumen. It can be suspected in such scenarios as elucidated in Table 6.

**Table 6:** Features on core needle biopsy portending a diagnosis of MBC MBC: metaplastic breast carcinoma

Sl No	Findings on core biopsy for suspicion of MBC	Corresponding histology of MBC
1.	Invasive breast carcinoma (low grade/high grade) exhibiting squamous differentiation	Adenosquamous carcinoma/squamous cell carcinoma (SCC)
2.	Pure SCC	SCC
3.	High-grade morphology exhibiting both epithelial and sarcomatoid areas	Sarcomatoid carcinoma/carcinosarcoma
4.	Atypical/malignant-looking spindle cell proliferations (resembling high-grade soft tissue sarcoma)	Sarcomatoid carcinoma/Spindle cell carcinoma
5.	Tumors exhibiting heterologous mesenchymal elements such as osteoid and/or chondroid	MBC with heterologous mesenchymal differentiation
6.	Spindle cell neoplasm, low grade	Fibromatosis like MBC

Immunohistochemistry (IHC) is an integral part of the diagnosis of MBC. In the situations mentioned in Table 6, especially in points (3), (4), (5), and (6), a diagnosis of MBC becomes plausible based on the evidence of epithelial differentiation by IHC analysis. Positivity of the tumor cells in the aforementioned instances, irrespective of the morphology, for pancytokeratin (PanCK) proves the epithelial nature and thus, a diagnosis of MBC can be proffered. The staining intensity can vary, and even a patchy expression is not to be ignored. High-molecular-weight cytokeratins (HMWCK)/basal cytokeratins, such as CK5/6 and 34beta12, are usually positive in MBC [19]. p63 is another important marker in the diagnosis of these cancers, with high sensitivity and specificity (86.7 % and 99.4%, respectively). p63 staining may be observed in both the epithelial and spindle cell components [20]. CD10 is commonly expressed in spindle cell carcinomas (94%); however, it is less frequently found in other types (0-71%). CK7 positivity is seen in around 30-60% of MBCs [21]. Notwithstanding the mention of these latter markers, the importance of PanCK positivity is paramount and is essential for the diagnosis of MBC.

Although fine needle aspiration cytology (FNAC) was not encountered in our study, it usually is an initial investigation performed for breast carcinomas, and the cytology features of MBC thus ought not to be overlooked. Clues for the diagnosis of MBC on FNAC are the presence of biphasic tumor cells with atypical spindle cells, atypical squamous cells, osteoclast-like giant cells, and/or matrix with or without a component of atypical ductal cells. However, it should be borne in mind that a cytologic diagnosis of MBC may not be attainable because of selective sampling of various pathological elements [8]. The significance of veracious recognition of MBC on core biopsy/FNAC lies in the fact that if misdiagnosed as non-epithelial malignancy, such as spindle cell neoplasm/sarcoma, there remains a high likelihood of the surgical management not including axillary nodal resection along with primary breast mass excision and thus being inappropriate.

IHC is an important tool not only for the diagnosis of MBC but also for management with regard to hormonal therapy. The vast majority (> 90%) of MBCs lack expression of ER, PR, and Her2/neu [8,21-23]. However, a significant atypical observation in our study was the striking hormonal receptor positivity (ER and/or PR) in 39.3% of cases (11 patients), whereas three cases (10.7%) were Her2/neu enriched. The histologic type varied among the hormone receptor-positive cases, SCC (4), SC (3), HGAS (2), and MCHMD (2). The three Her2/neu positive cases belonged to HGAS (2) and SCC (1). Noteworthy is the fact that SCC demonstrated positivity for hormone receptors as well as Her2/neu. The above findings underline the significance of evaluating hormone receptor profiles in MBCs. In positive cases, hormonal therapy is advisable to be administered. Our study also divulged the relatively less percentage of disease progression on NACT and the preponderance of disease-free status in the TNMBC subgroup when collated with the NTNMBCs. The observations documented by Lim KH et al. somewhat resonate with our study, although the percentage in NTNMBC is lower (19.6%) versus that of TNMBC (80.4%) [24]. Lim KH et al. indicated that the NTNMBC group had a poor prognosis compared with the TNMBCs, which is contrary to what has been reported in patients with IBC NST; NTNMBC has a poorer prognosis in overall survival (OS) than TNMBC, and this triple negativity is a good prognostic factor in MBC. Also, after distant metastasis, NTNMBC tends to progress rapidly, which could lead to a significant difference in OS between the two subgroups [24]. However, the sanctity of the aforementioned facets and mechanisms underlying these results need to be ascertained by long-term studies.

Due to the rarity of this tumor, there are no standard guidelines for optimal management, and treatment is similar to IBC NST. Surgery is the mainstay of treatment, and treating MBC is challenging owing to the poor response to NACT and the absence of novel targeted therapies [25]. The majority of the patients (67.9%) in our study required mastectomy rather than breast conservation (21.4%) because of the larger size of the tumor at presentation and poorer response to conventional chemotherapy. One patient developed solitary liver metastasis on NACT; she was treated with curative intent (liver metastasectomy) during the primary surgery. Axillary staging in MBC is similar to IBC NST with axillary sampling in N0 axilla and axillary dissection in node-positive axilla. Due to the low reported rate of axillary lymph node involvement, for accurate nodal staging, Murphy et al. recommended the utility of axillary ultrasound/FNAC at diagnosis followed by sentinel lymph node surgery in MBC [26]. The prospect of lymph node (LN) involvement oscillates with the histopathologic subtype of MBC; 10-15% of patients with SCC have LN metastases at presentation while up to 25% of chondro-osseous element-containing MBCs are LN-positive. Murphy et al. reported that patients with squamous cell variants of MBC have the highest rate of LN involvement while simultaneously highlighting the lack of statistical significance across all histologic subtypes in this aspect [26]. In our study, similarly, SCC exhibited a higher incidence of LN involvement, although, in numerical comparison, SC scored over the others.

Erjan et al. reported that in 33 of the 81 MBC patients (~ 40%) who received NACT, 14 patients (42.4%) had disease progression, and only two patients (6%) achieved a pathological complete response [15]. In our series, 14 patients (50%) received NACT, out of which five patients (35.7%) harbored stable disease, another five (35.7%) manifested disease progression, four (28.6%) illustrated partial response, and none evinced complete response. Wong et al. illustrated that there was a poor response or disease progression on NACT in patients with MBC and suggested that NACT should be reserved for patients with inoperable MBC [27]. Adjuvant RT was administered to 16 patients (57.1%) in our cohort. The role of RT post-mastectomy is limited. Tseng et al. suggested that RT should be included in the multimodality management for MBC patients undergoing BCS and those patients with tumors > 5 cm or > four metastatic axillary lymph nodes undergoing mastectomy [28]. In this group of patients, adjuvant RT provides statistically significant overall survival (OS) and disease-specific survival benefit. Patients undergoing mastectomy with tumors < 5 cm or < four metastatic axillary lymph nodes derived no benefit from RT [28]. Despite low rates of axillary involvement, MBC has a high potential for distant metastases via the hematogenous route (mostly lung and bone). Song et al. recorded 18.1% of locoregional recurrence and 41.8% of distant metastasis [13] while the detection of the same was lower in our study (3.6% locoregional recurrence and 32.1% distant metastasis). The mortality incidence in our study was 39.3% (11/28) on a median follow-up of 13.2 months. MBC has a worse prognosis than IBC NST and TNBC; the five-year overall survival rate for MBC was 54.5% compared to 85.1% for IBC NST and 73.3% for TNBC [13].

## Conclusions

In summary, although MBC is a rare malignancy, it should be a consideration when encountering patients with a rapidly growing breast lump. Core biopsy often fails to diagnose MBC and a high index of suspicion while confronting an atypical morphology not fitting into conventional IBC or malignant phyllodes tumors can help clinch the diagnosis. IHC is an invaluable tool in this diagnostic pursuit. A notable detection in our study is the sizeable number of hormone receptor-positive MBCs (39.3%). The majority (53.6%) are TNBC; however, unlike TNBC, their response to NACT is dismal. Upfront surgery is preferred whenever feasible. NACT may only select patients with better tumor biology; nonetheless, there is an increased risk of progression on NACT. Patients who unveil disease progression on NACT should be re-assessed for distant metastasis before offering surgery. Owing to it being a recherche entity, the smaller sample size can be a limiting factor in extrapolating the findings of our study. Thus, a larger series of patients is required to conduct clinical trials and to discover molecular targets for the identification of subgroups of the disease, so that potential tumor-specific targeted therapies can be developed and prognosis be enhanced.
